# Between the gate and the stars

**DOI:** 10.1093/oncolo/oyag408

**Published:** 2026-10-12

**Authors:** Ettore D’Argento, Alessandro Scala

**Affiliations:** Medical Oncology Department, Comprehensive Cancer Center, Fondazione Policlinico Universitario Agostino Gemelli IRCCS, Rome, Italy; Medical Oncology Department, Comprehensive Cancer Center, Fondazione Policlinico Universitario Agostino Gemelli IRCCS, Rome, Italy

*“Lasciate ogne speranza, voi ch’intrate”*

*Abandon hope, all ye who enter here.*

—Dante Alighieri, Inferno, Canto III

It was a Tuesday in early July. Outside the window, Rome shimmered. Through the haze, distant and weightless in the trembling air, the dome of St. Peter’s was just visible on the horizon. A hot breeze moved through the room, carrying the unrelenting drone of cicadas from the park surrounding the hospital.

Marco was sixty-nine, a former electrician from the suburbs of Rome. He had relapsed small-cell lung cancer. Thirteen months earlier, we had been through platinum—etoposide and durvalumab—the initial response, the brief and merciful shrinkage, then the return. As at every appointment for a year, Marco asked whether the new drug had finally reached our clinic. He struggled to pronounce it—tarlatamab—as though the word were foreign territory. Each time, the question came after a pause I had learned to dread. “When will I be able to receive it?” As I had every time before, I told him I was working on it.

Elena was always with him. Sixty-seven, a retired flight attendant, she had been diagnosed with metastatic pancreatic adenocarcinoma shortly after her husband’s diagnosis. She was followed at an oncology center in the southern outskirts of Rome. They managed their grandchildren, trading appointments, organizing days with quiet precision. Each was the other’s caregiver.

Elena had been through gemcitabine, nab-paclitaxel, and FOLFIRINOX. She was not given to complain. That afternoon, with her hands folded in her lap, she said, “I know you are not my doctor directly. But since you work at a larger hospital, I hope you will forgive me for asking.” She reached into Marco’s folder and produced a newspaper clipping. The headline proclaimed that daraxonrasib had achieved the first major breakthrough after decades of therapeutic stagnation in pancreatic cancer. She could not pronounce the name of the drug. After a pause, she looked at me: “Will I ever be able to try it?” I told her—not entirely concealing my discomfort—it would become available as soon as possible.

Those two questions, and my two answers, stayed with me. I began to wonder whether “I am working on it” was honest advocacy or merely the gentlest language for my own powerlessness.

When I think about this suspension—between what science has achieved and what patients can reach—I return to Dante.

The *Divine Comedy* is, at its core, a poem about movement. Hell is immobility: fixed sentences, irreversible condemnation. Purgatory is ascent: effort, possibility, purpose slowly recovered. Paradise ends in the vision of *“l’amor che move il sole e l’altre stelle”*—the love that moves the sun and the other stars. In Dante, hope coincides always with the possibility of advancing.

For patients with relapsed small-cell lung cancer, the journey has long begun near the gate of the Inferno. Tumors shrink, families breathe, then relapse arrives. Tarlatamab has altered that symbolic geography—offering more than a drug; offering a breach in the architecture of inevitability. The gate is not a wall but a threshold. And thresholds can be crossed.

Patients with pancreatic adenocarcinoma inhabit a different Dantesque realm: not the Inferno, but Limbo. In Canto IV, souls are not tortured by flames. Their suffering is quieter—desire without fulfillment, knowledge without arrival, longing without completion. For decades, pancreatic cancer lived in exactly that place. Daraxonrasib has begun to illuminate a slope where the darkness itself had long made everything still. It does not promise the summit. But it shows, at last, that the mountain can be climbed.

Marco and Elena stand at these thresholds. The science has moved. Yet the distance between what the data show and what my patients can receive cannot be closed by evidence alone.

In Dante’s poem, no one crosses the dark wood alone. Virgil appears not as a savior but as a guide—unable to enter Paradise, yet able to interpret the darkness and keep Dante moving. I think of Virgil often in the clinic. I am not a redeemer. I cannot command time nor promise stars. But I accompany patients through territories they never chose to enter, translate data into meaning, and point toward openings in the dark. And sometimes—as I did that Tuesday in July—I stand before a door I can see but cannot open.

Powerlessness is not quite the right word. What that afternoon imposed was something more specific: a moral condition particular to this moment in oncology—one that calls not for resignation, but for witness. It is measured in clinic rooms, in pauses before unanswerable questions, in the discomfort we cannot entirely conceal.

Our patients ask for very little. Not miracles, not promises beyond the evidence. Only that when science has found a path, the systems around it allow them to walk it.

Dante ends his journey through darkness with a line I have carried since before I became a physician:*“E quindi uscimmo a riveder le stelle”*—And we came out to see again the stars.

For Marco. For Elena. For all the patients waiting between the gate and the stars.

Do not leave them there.

